# Role of plant metabolites in the formation of bacterial communities in the rhizosphere of *Tetrastigma hemsleyanum*

**DOI:** 10.3389/fmicb.2023.1292896

**Published:** 2023-12-15

**Authors:** Yuqing Huang, Hongliang Hu, Erkui Yue, Wu Ying, Tianxin Niu, Jianli Yan, Qiujun Lu, Songlin Ruan

**Affiliations:** ^1^Institute of Crop Science, Hangzhou Academy of Agricultural Sciences, Hangzhou, China; ^2^College of Life Sciences, China Jiliang University, Hangzhou, China; ^3^Hangzhou Agricultural and Rural Affairs Guarantee Center, Hangzhou, China

**Keywords:** *Tetrastigma hemsleyanum*, Sanyeqing, rhizosphere microbiome, metagenome, microbial community structure, metabolite profiling, bioactive constituents

## Abstract

*Tetrastigma hemsleyanum* Diels et Gilg, commonly known as Sanyeqing (SYQ), is an important traditional Chinese medicine. The content of bioactive constituents varies in different cultivars of SYQ. In the plant growth related researches, rhizosphere microbiome has gained significant attention. However, the role of bacterial communities in the accumulation of metabolites in plants have not been investigated. Herein, the composition of bacterial communities in the rhizosphere soils and the metabolites profile of different SYQ cultivars’ roots were analyzed. It was found that the composition of microbial communities varied in the rhizosphere soils of different SYQ cultivars. The high abundance of *Actinomadura*, *Streptomyces* and other bacteria was found to be associated with the metabolites profile of SYQ roots. The findings suggest that the upregulation of rutin and hesperetin may contribute to the high bioactive constituent in SYQ roots. These results provide better understanding of the metabolite accumulation pattern in SYQ, and also provide a solution for enhancing the quality of SYQ by application of suitable microbial consortia.

## 1 Introduction

*Tetrastigma hemsleyanum* Diels et Gilg (Sanyeqing, SYQ) is a green vine species belonging to the Vitaceae family (Huang et al., 2005). It is distributed mainly in the southern part of China, such as Zhejiang, Guangxi and Guizhou Provinces. Although the whole SYQ plant can be used as herbal medicine, the tuberous roots are the most utilized part for medicinal use. SYQ contains many bioactive constituents, including flavonoids, phenols, polysaccharides, amino acids, terpenes and alkaloids (Zhu et al., 2020). Studies have shown that SYQ possesses many pharmacological properties, such as antitumor and antibacterial properties (Chen et al., 2019; Sun et al., 2021a). Flavonoids and phenols were the representative ingredients in SYQ, metabolites profiling were applied to analysis the compositions of flavonoids, polyphenols, polysaccharides and other ingredients. For example, 23 and 21 phenolic acids were found in the aerial part and root of SYQ, respectively. The high enrichment of phenolic acids in SYQ suggests their critical role in plant growth and development (Tao et al., 2021). In addition, total flavonoids in SYQ were found to have anti-lung cancer activity and protect from acute lung injury in mice, providing new pathways for development of anti-tumor and anti-inflammatory drugs (Aditya and Ajay, 2017). Thus, breeding SYQ cultivars with high bioactive constituents is important for increasing its economic value.

Microbes present in the soil are involved in the nutrient cycling, soil structure formation, and response to abiotic and biotic stress (Mayak et al., 2004; Brussaard et al., 2007). Plant growth has also been reported to be regulated by the interactions between plants and soil microbiome (Sasse et al., 2018). The performance of plants depends on the genetic features, as well as the microbial communities in rhizosphere soil. Numerous investigations have discovered that plant domestication significantly affects the rhizosphere microbiome (Sun et al., 2021b). Root exudates secreted during plant growth play an important role in the formation of rhizosphere microbial communities (Pascale et al., 2020). The nutrient availability in rhizosphere has been found to be affected by root exudates, such as amino acids, sugars, flavonoids and lipids (Bais et al., 2006). Thus, soil microbiomes play a crucial role in regulating the plant growth and agricultural yield (Schlaeppi et al., 2014; Iannucci et al., 2017).

Recent studies have showed that the microbiome profiles in rhizosphere soil are shaped by host genotype, domestication, and plant development process (Bulgarelli et al., 2012, 2015). The composition of metabolites in plants was found to be influenced by soil characteristics (Hamoud et al., 2022). Wilt resistance of tomato improved after the introduction of *Flavobacterium* sp. in rhizosphere (Kwak et al., 2018). Similar correlations between disease resistance and soil microbiome were observed in some other plants (She et al., 2017; Xiong et al., 2017; Shi et al., 2019; Wang et al., 2022). The relationship between the plants and the surrounding environment is complex. Therefore, proper management of soil microbial communities is necessary to improve the agricultural production. In-depth research on soil microbiome can provide new insights regarding the impact of microbiomes on plant growth.

In the past decade, 16S rRNA gene library construction has been used as a key method for soil microbial characterization. With the rapid developments in next-generation DNA sequencing, metagenomics analysis has emerged as a novel, cost-effective, and user-friendly tool for genome analysis (Thompson et al., 2017). The application of metagenomics has helped in improving the understanding of microbial diversity and abundance. The analysis of microbial genome sequences is an efficient way to decipher the unknown microbial species (Woodcroft et al., 2018). It provides a clear understanding of soil microbial dynamics at a genomic scale.

Despite the studies regarding correlations between plant growth and soil microbiomes, it is still unclear how specific rhizosphere microbiome shape the metabolite profiles in SYQ. In this study, the differences in metabolite profiles of different SYQ cultivars have been analyzed and compared with the microbial communities in the soil surrounding them. Our finding provides insights into the microbiome-metabolites-bioactivity regulation system of SYQ roots.

## 2 Materials and methods

### 2.1 Field experiment and sampling

Four SYQ cultivars were planted in early May 2019 in Hangzhou Academy of Agricultural Sciences. The detailed information of every cultivar was listed in Supplementary Table S1. Every cultivar was cultivated in triplicates and in accordance with local agronomic practices. Samples (rhizosphere soils and roots of cultivars) were collected in May 2022 (3 years after planting). The rhizosphere soils were sifted through a 2-mm mesh and stored at −80°C immediately. The roots of different SYQ cultivars were collected for analysis and extraction of metabolites.

### 2.2 Determination of total polyphenol and flavonoids

The quantification of ethanol-soluble extract was done according to the Chinese Pharmacopoeia method (2020). Total flavonoids content was measured as described by Zheng et al. (2018). Total polyphenol content was measured as described by Sun et al. (2013).

### 2.3 Metagenomic sequencing and assembly

Genomic DNA were extracted from the rhizosphere soil samples and paired-end libraries were constructed. The libraries were pooled and sequenced using Illumina sequencing platform. Low-quality metagenomic reads (length < 40 bp, N bases >10 bp, overlap with adapters >15 bp) were removed. MEGAHIT software (v1.0.4-beta) was applied to analyze the clean data (Nielsen et al., 2014; Li et al., 2015). MetaGeneMark^1^ (V3.05) was used for the prediction of ORFs. Subsequently, CD-HIT^2^ software (V4.5.8) was applied for the acquisition of non-redundant genes (Fu et al., 2012). The gene abundance of obtained clean data was calculated by use of Bowtie2. Afterwards, DIAMOND^3^ software (V0.9.9.110) was used for the gene annotations by comparing the non-redundant gene catalogues with NCBI NR database and KEGG database (Buchfink et al., 2015).

### 2.4 Metabolic profiling

The roots of SYQ were grounded in liquid nitrogen and resuspended in 80% methanol. The suspensions were centrifuged at 12,000 rpm for 10 min at 4°C. Afterwards, the supernatant was diluted and transferred to a new tube. The sample extracts were analyzed by ultra-high performance liquid chromatography (UHPLC) (Thermo Fisher, Germany) coupled with an Orbitrap Q Exactive™ HF mass spectrometer (Thermo Fisher, Germany). Hypesil Gold column (100 × 2.1 mm, 1.9 μm) was used for the separation of compounds in extracts. The eluents in positive polarity mode were eluent A (0.1% formic acid in water) and eluent B (methanol), while eluents in negative polarity mode were eluent A (5 mM ammonium acetate, pH 9.0) and eluent B (methanol). The detailed parameters were set according to Li et al. (2023). The mass fragmentations were blasted in the HMDB,^4^ METLIN^5^ and KEGG^6^ databases. SIMCA-P V12.0.0 Demo (Umetric, Umea, Sweden) was used for principal components analysis (PCA). Cluster analysis was conducted by R.

## 3 Results

### 3.1 Quantification of flavonoid content and total phenolic content in SYQ roots

**Figure 1:**
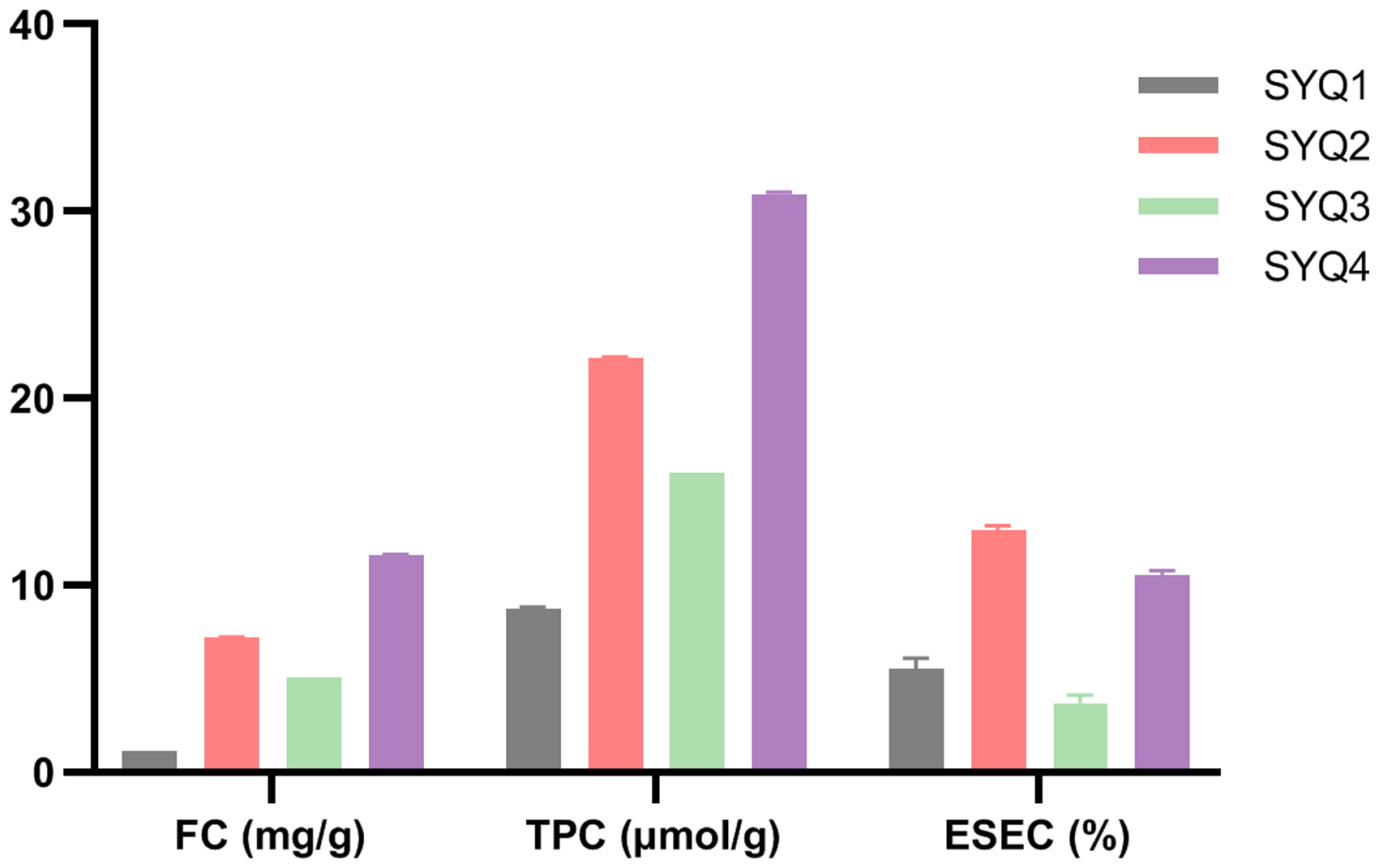
FC, TPC, and ESEC in the roots of four cultivars. FC, flavonoid content; TPC, total phenolic content; ESEC, ethanol-soluble extractive content.

The measured flavonoid content (FC, mg/g), total phenolic content (TPC, μmol/g), and ethanol-soluble extractive content (ESEC, %) in SYQ roots have been shown in Figure 1. FC, TPC, and ESEC in SYQ1 and SYQ3 were significantly lower than the respective contents in SYQ2 and SYQ4. This indicated that the bioactive constituents of SYQ2 and SYQ4 were higher than SYQ1 and SYQ3.

### 3.2 Bacterial community composition in rhizosphere soils related to different SYQ cultivars

**Figure 2:**
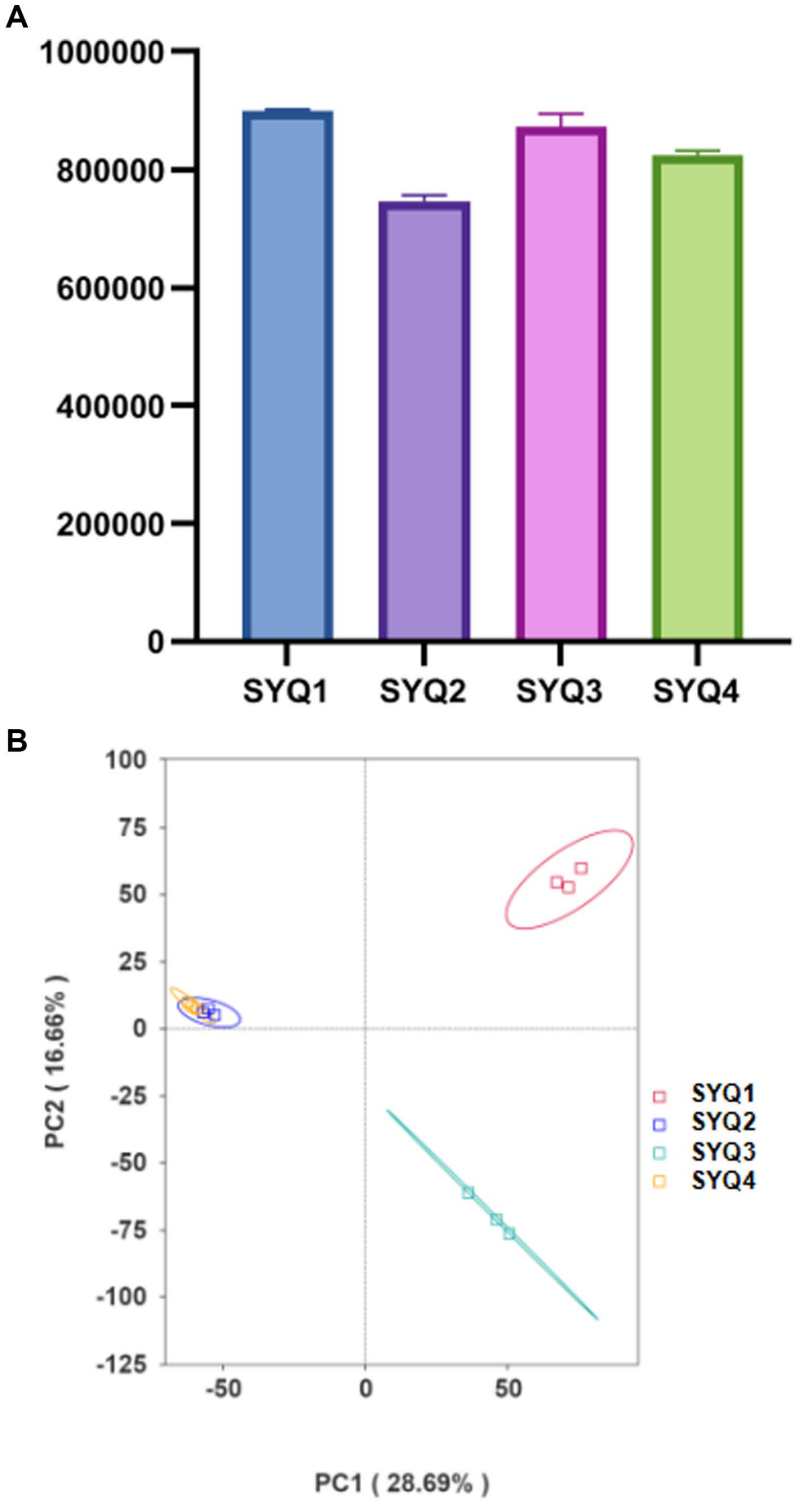
Metagenomics analysis of different soil samples. **(A)** The number of non-redundant genes in different cultivars. **(B)** PCA of bacterial composition in four soil samples.

In this study, a total of 77,785 raw reads were identified by Illumina sequencing. After filtering, 77,741 clean reads were obtained and the effective rate was 99.94%. A total of 1,380,063,369 nucleotide bases were identified and the N50 was 669. The maximum contig was 86,850 bp (Supplementary Table S2). Figure 2A showed the number of non-redundant genes in the samples. Among the four SYQ cultivars, the highest number of non-redundant genes was found in SYQ1, while the lowest number was in SYQ2.

Principal component analysis (PCA) showed that SYQ2 and SYQ4 were clearly separated from SYQ1 and SYQ3 at species level (Figure 2B). PC1 explained 28.69% of the variations in microbial communities. This suggested that the bacterial community structure of SYQ2 and SYQ4 were similar and different from SYQ1 and SYQ3. The cluster analysis showed that the bacterial communities in the rhizosphere soils of four cultivars were different from each other.

**Figure 3:**
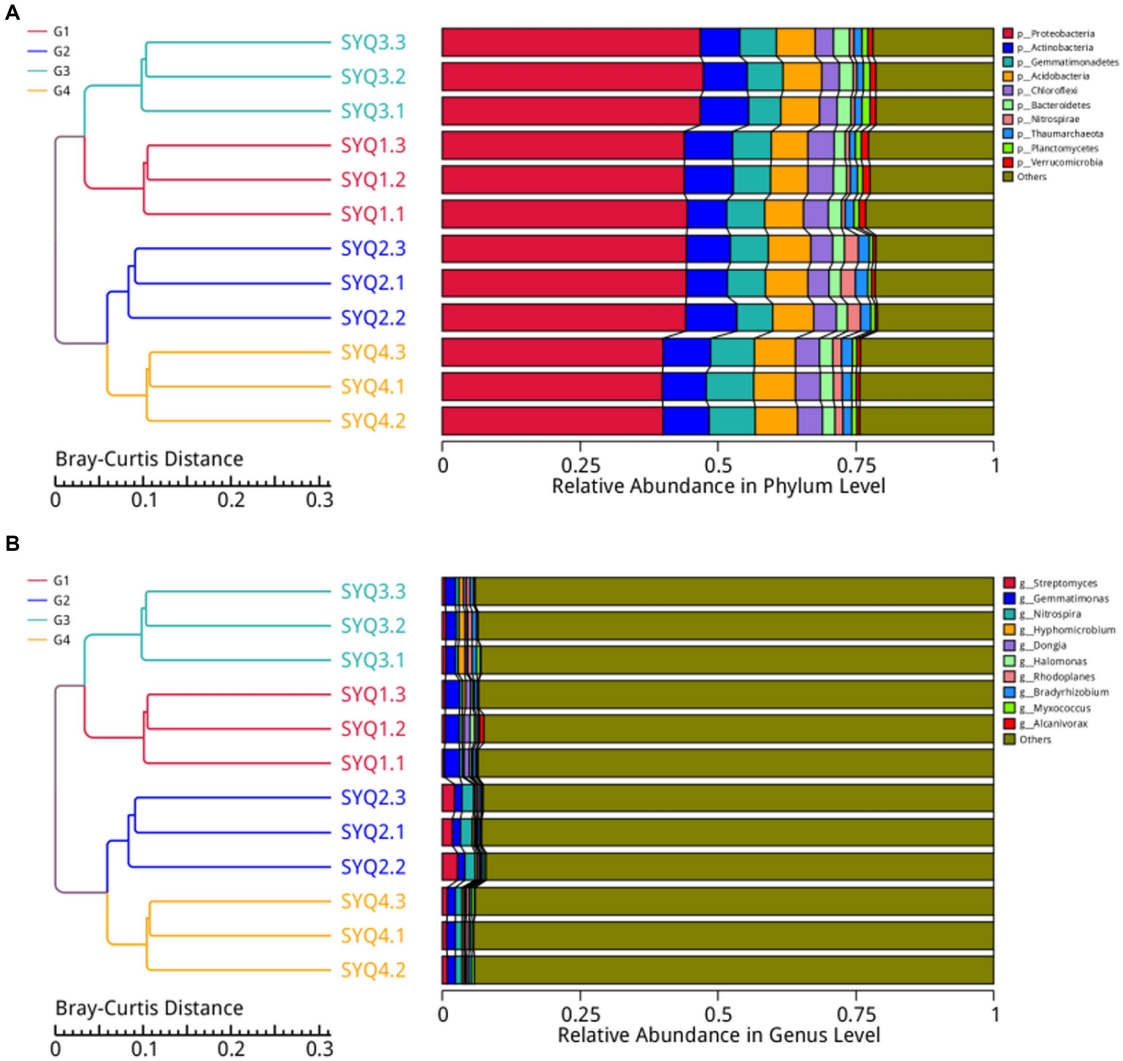
Bacterial structure analysis of different soil samples. **(A)** Bacterial structure at phylum level in four soil samples. **(B)** Bacterial structure at genus level in four cultivars.

The dominant bacterial phyla in the four cultivars were Proteobacteria, Actinobacteria, Gemmatimonadetes, Acidobacteria, Chloroflexi, Bacteroidetes, Nitrospirae, Thaumarchaeota, Planctomycetes, and Verrucomicrobia (Figure 3A). These dominant phyla accounted for 75% of the entire bacterial community, while Proteobacteria was the most abundant phyla. To gain a deeper insight into the bacterial abundance in different cultivars, microbial community analysis at the genus level was applied. The main genera in four cultivars were *Streptomyces, Gemmatimoonas, Nitrospira*, and *Hyphomicrobium* (Figure 3B). The abundances of *Streptomyces* and Nitrospira were higher in SYQ2, compared to other three cultivars. In SYQ1, abundance of *Gemmatimoonas* was the highest.

**Figure 4:**
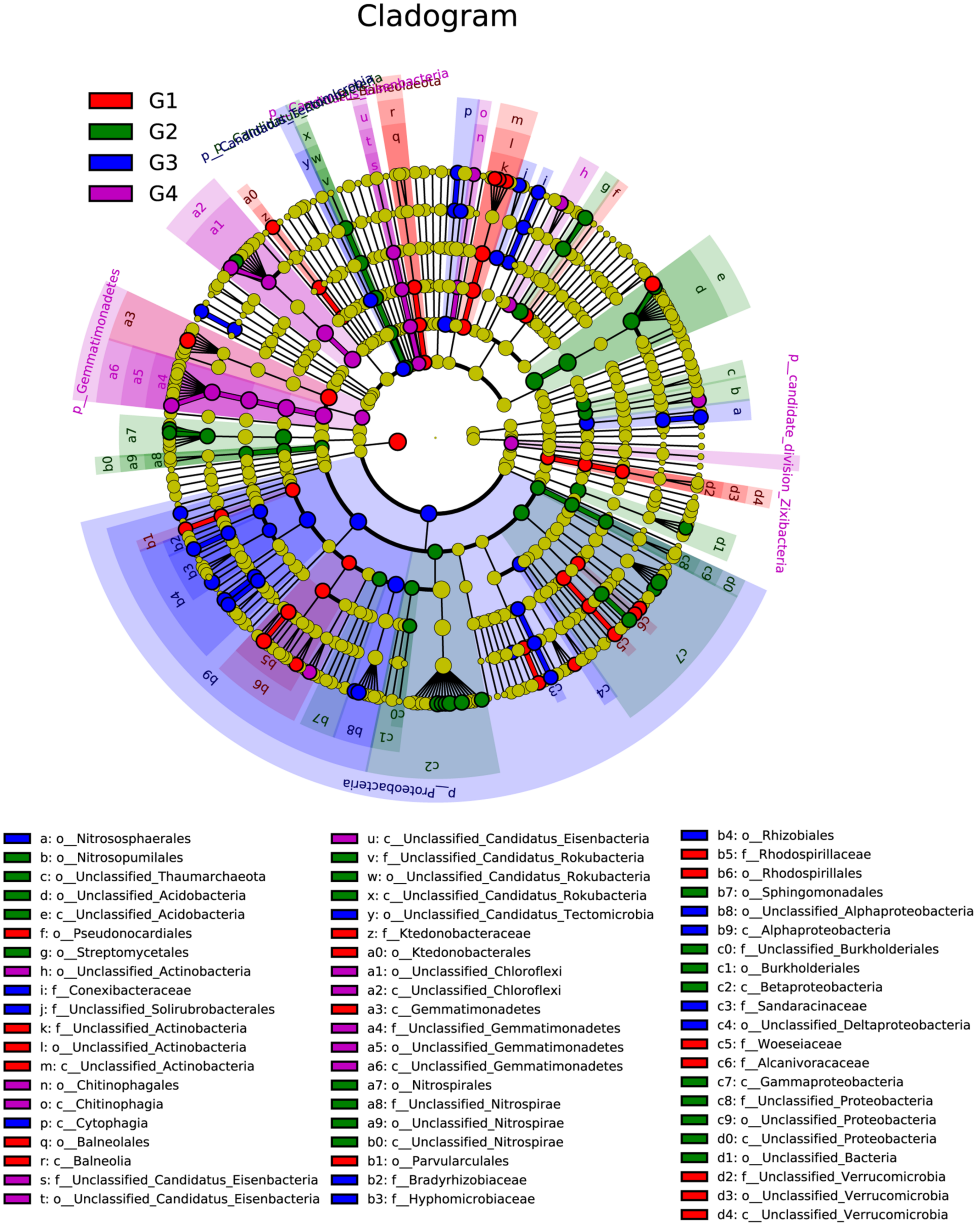
LEfSe analysis showing the different taxon among different SYQ cultivar rhizospheres bacterial communities. Different colored regions represent different culitvars (red for SYQ1, green for SYQ2, blue for SYQ3 and purple for SYQ4) and the diameter of each circle is proportional to the relative abundance of the taxon. The inner to outer circle represents the level of the phylum to the genus.

To gain more information on the rihizosphere bacterial communities of different cultivars, LEfSe was used to identify the differentially abundant communities among different cultivars (Figure 4). According to the result of LEfSe analysis of the rhizosphere community, 61 distinctly abundant taxa were discovered in different cultivars. Among the 61 bacteria taxa, there were 17, 21, 12, and 11 taxa were enriched in SYQ1, SYQ2, SYQ3, and SYQ4, respectively.

**Figure 5:**
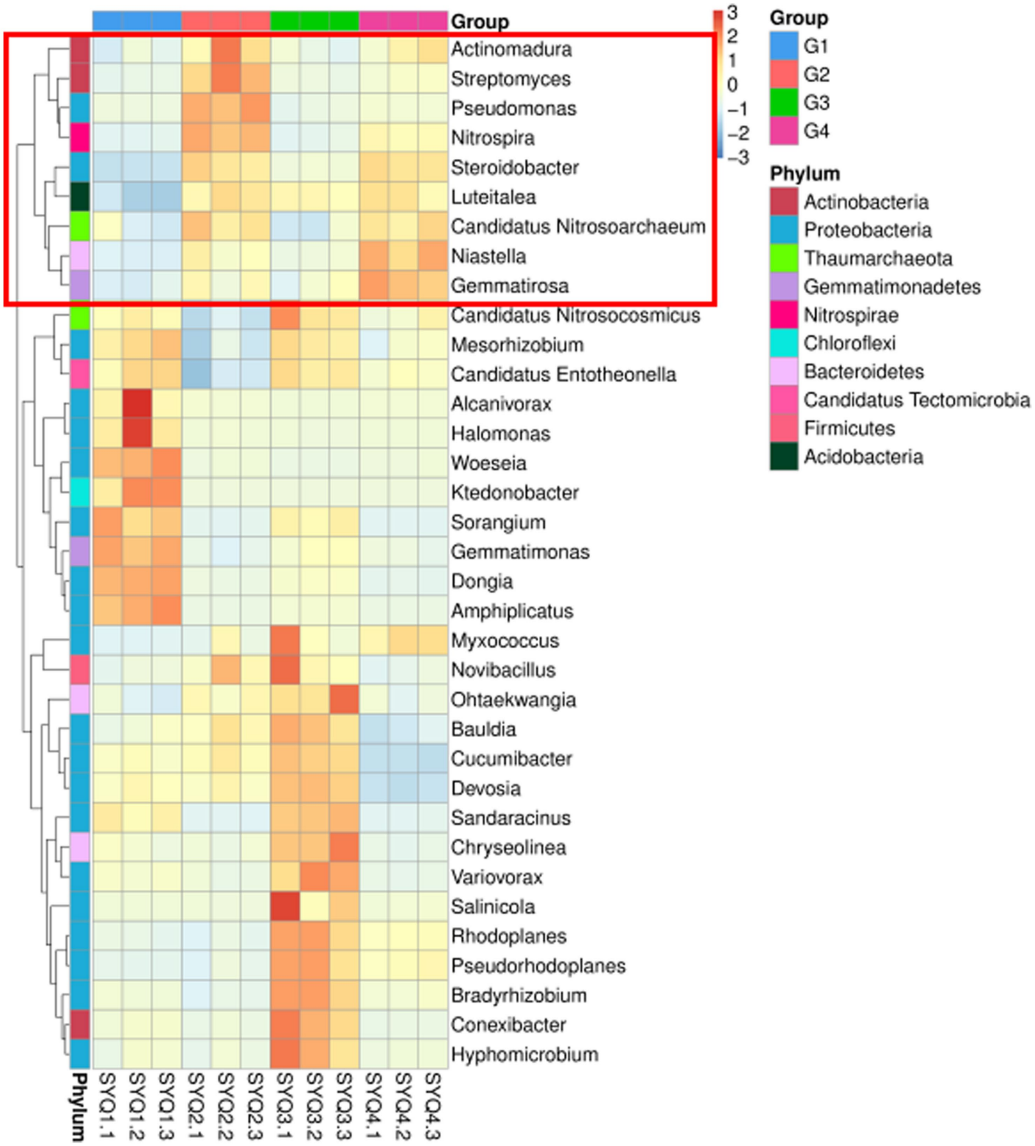
Clustering heatmap of bacterial communities at genus level. G1, SYQ1; G2, SYQ2; G3, SYQ3; G4, SYQ4. The bacterial genus framed in red box had a higher abundance in SYQ2 and SYQ4.

The abundance of *Actinomadura, Streptomyces, Pseudomonas, Nitrospira, Steroidobacter, Luteitalea, Candidatus, Nitrosoarchaeum, Niastella* and *Gemmatirosa* was higher in SYQ2 and SYQ4 (Figure 5). The accumulation of these bacterial communities may be related to the bioactivity of the corresponding cultivars.

**Figure 6:**
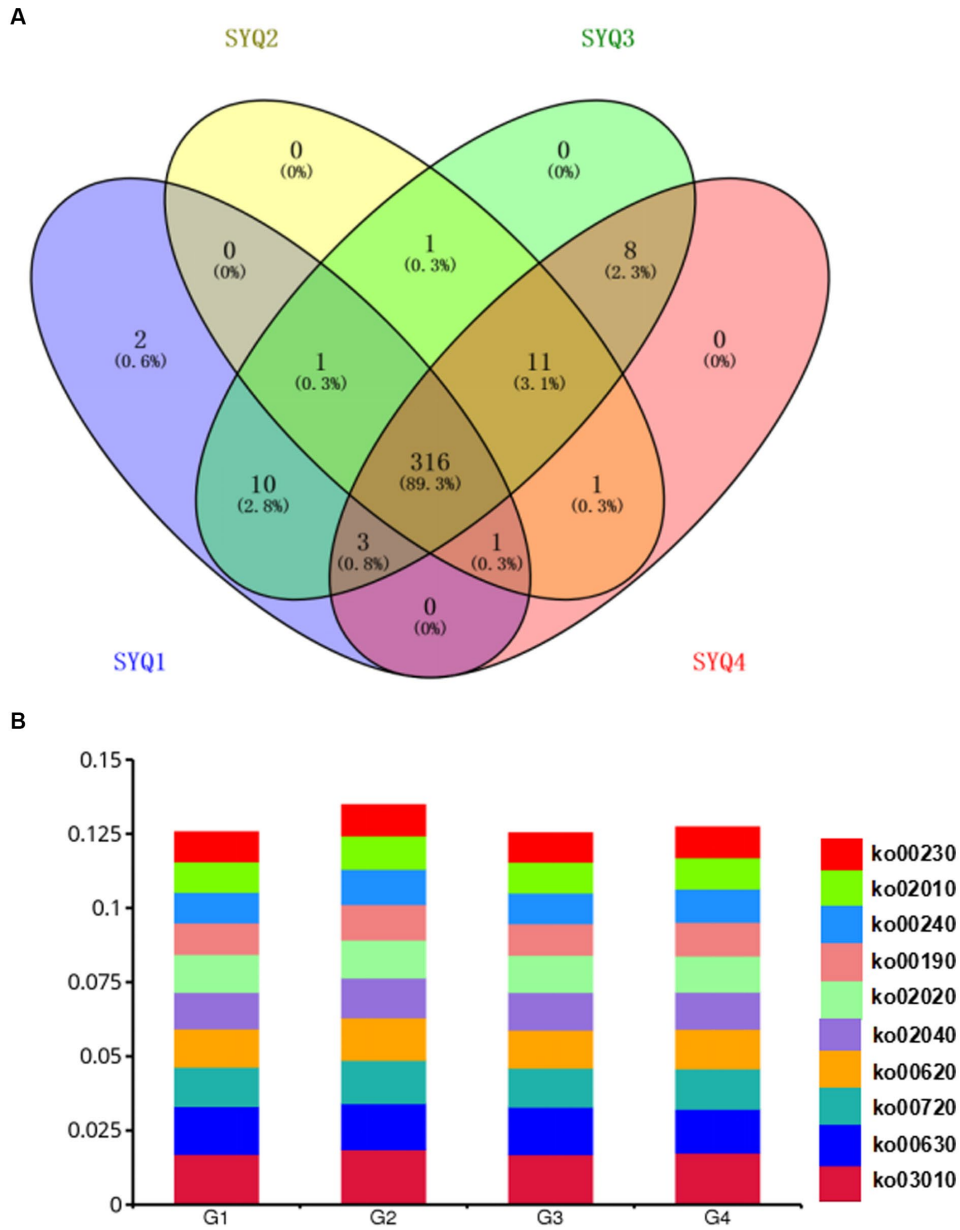
KEGG analysis of the genes identified by metagenomics analysis. **(A)** Venn diagram of shared level 3 KEGG pathways among the rhizosphere soils of four cultivars. **(B)** The dominant KEGG pathways of different cultivars. G1, SYQ1; G2, SYQ2; G3, SYQ3; G4, SYQ4.

The identified genes were annotated according to KEGG database and mapped onto 353 Level 3 KEGG pathways. As shown in the Venn diagram (Figure 6A), 316 pathways were shared by the four cultivars. Only 2 pathways, i.e., spliceosome and relaxin signaling pathway were uniquely abundant in SYQ1. The dominant pathways were purine metabolism (ko00230), ABC transporters (ko02010), pyrimidine metabolism (ko00240), oxidative phosphorylation (ko00190) and two-component system (ko02020) (Figure 6B).

### 3.3 Metabolic profiling of different SYQ cultivars

**Figure 7:**
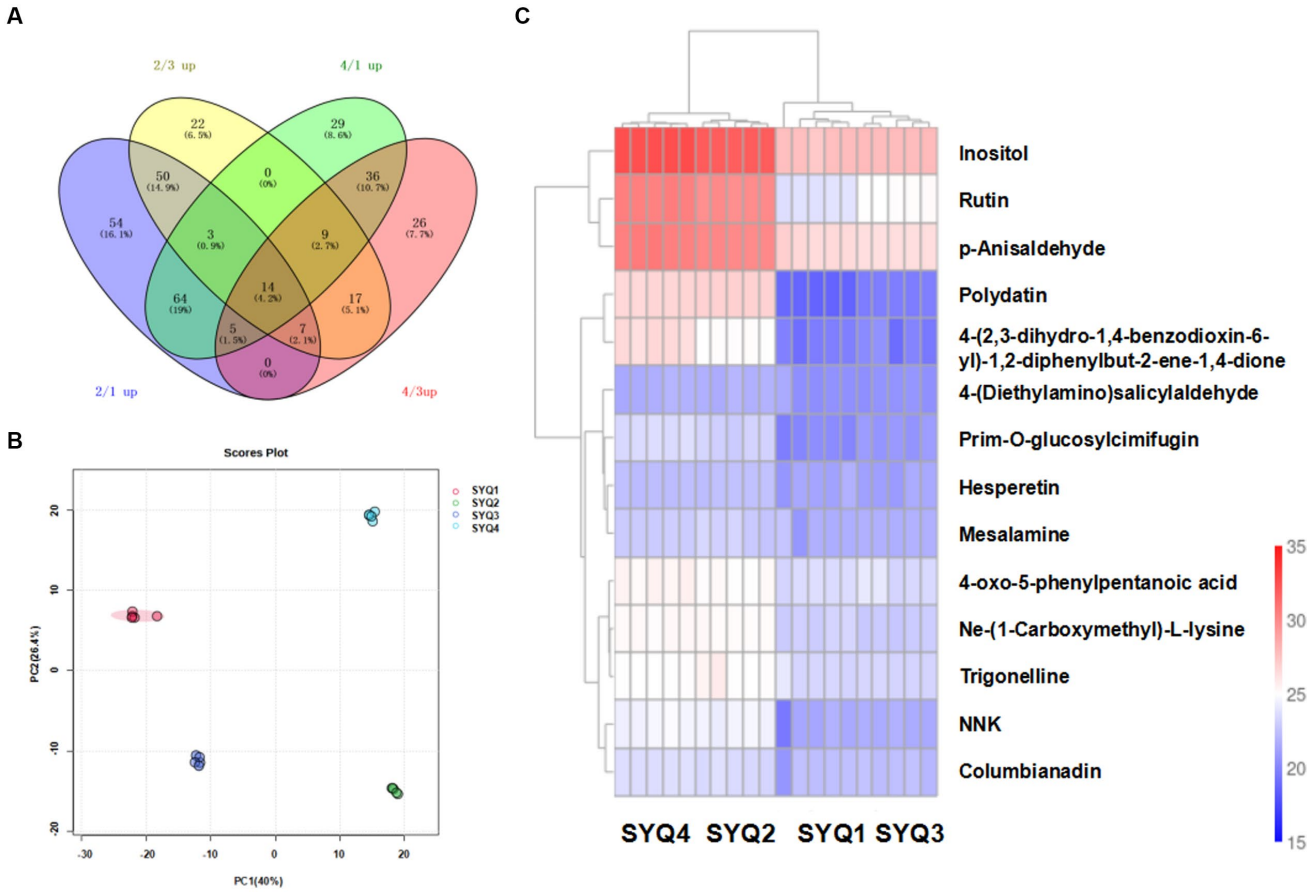
Metabolites profiles of different roots samples. **(A)** Venn diagram of shared metabolites in four cultivars. **(B)** PCA of metabolites in four cultivars, and **(C)** Clustering heatmap of metabolites in four cultivars.

UHPLC–MS/MS (Ultra-high-performance liquid chromatography mass spectrometry) analyses were performed to determine the metabolites profiles of the four SYQ cultivars. Total 767 metabolites were detected and quantified in the extracts of cultivars (Figure 7A). Metabolites with more than 1.5-fold change and *p* value of less than 0.05 were defined as differentially accumulated metabolites (DAMs).

PCA showed that the metabolite profiles of SYQ1 and SYQ3 were clearly separated from the metabolite profiles of SYQ2 and SYQ4 (Figure 7B). PC1 and PC2 explained 42.33% and 26.26% of the total variance in metabolite profiles, respectively. Hierarchical clustering analysis of the metabolites in different cultivars showed that the repeatability within the sample groups were good. SYQ 1 and SYQ3 were grouped in the same cluster, while SYQ2 and SYQ4 were grouped together in the same cluster (Figure 7C).

According to the Venn diagram, there were 14 commonly upregulated DAMs in SYQ2 and SYQ4, compared to SYQ1 and SYQ3. These DAMs included alkaloids, amino acids, flavonoids, organic acids, sugars and other metabolites. The two upregulated flavonoids were rutin and hesperetin. The distinctive accumulation patterns of these metabolites may account for the differences in bioactivities among different groups.

**Figure 8:**
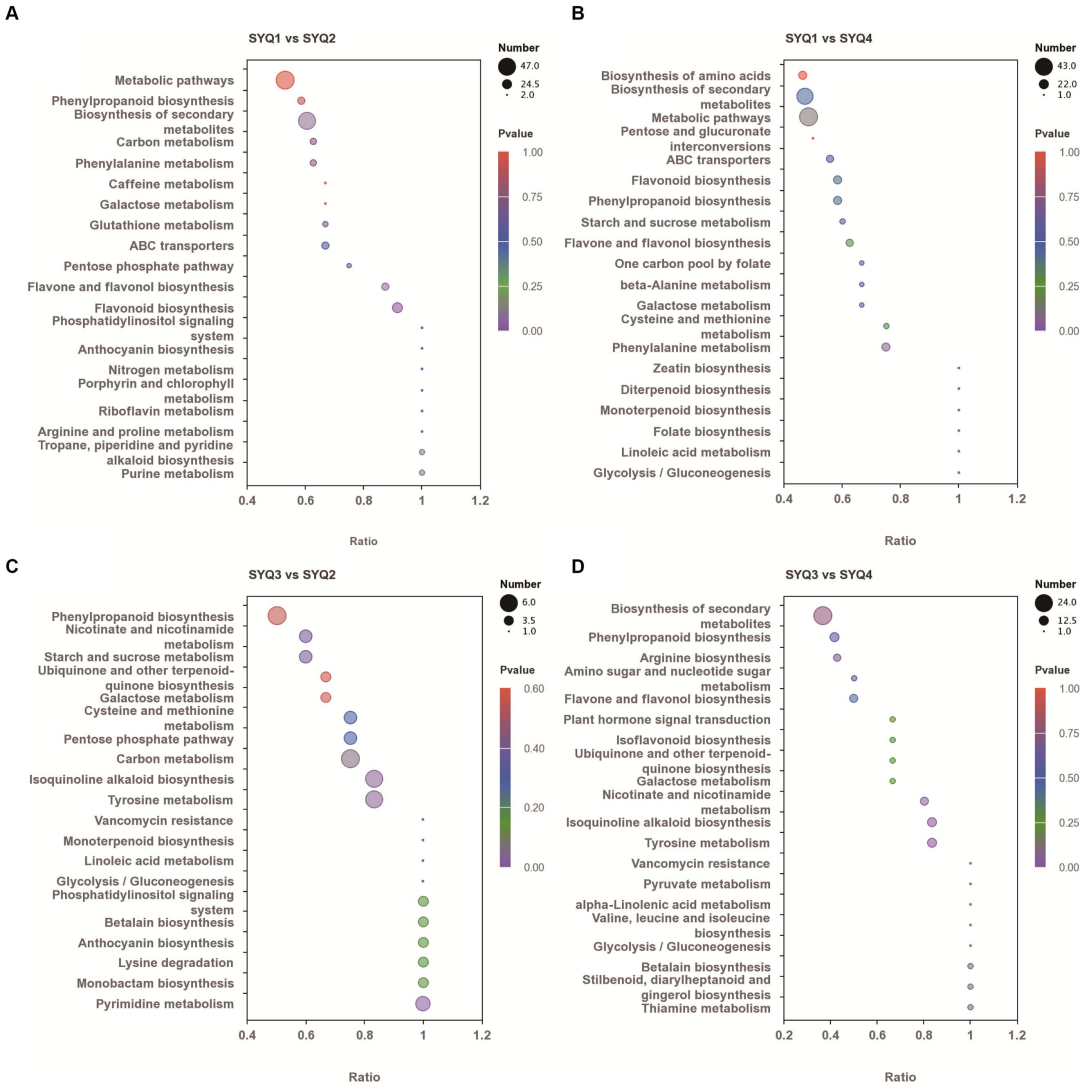
KEGG pathway enrichment analysis of differentially accumulated metabolites in four cultivars: **(A)** analysis between SYQ1 and SYQ2, **(B)** analysis between SYQ1 and SYQ4, **(C)** analysis between SYQ3 and SYQ2, and **(D)** analysis between SYQ3 and SYQ4.

KEGG enrichment analysis was performed on DAMs of different cultivars (Figure 8). The DAMs in SYQ1 and SYQ2 were mainly involved in metabolic pathways, biosynthesis of secondary metabolites, flavonoid biosynthesis, and flavone and phenylpropanoid biosynthesis. These metabolic pathways were also identified in SYQ1 and SYQ4. Phenylpropanoid biosynthesis, carbon metabolism, tyrosine metabolism, and isoquinoline alkaloid biosynthesis were significantly enriched in SYQ3 and SYQ2. While the DAMs in SYQ4 and SYQ2 were mainly related to biosynthesis of secondary metabolites, tyrosine metabolism, phenylpropanoid biosynthesis and isoquinoline alkaloid biosynthesis.

### 3.4 Correlation analysis of the abundance of bacterial community and metabolites profiles

**Figure 9:**
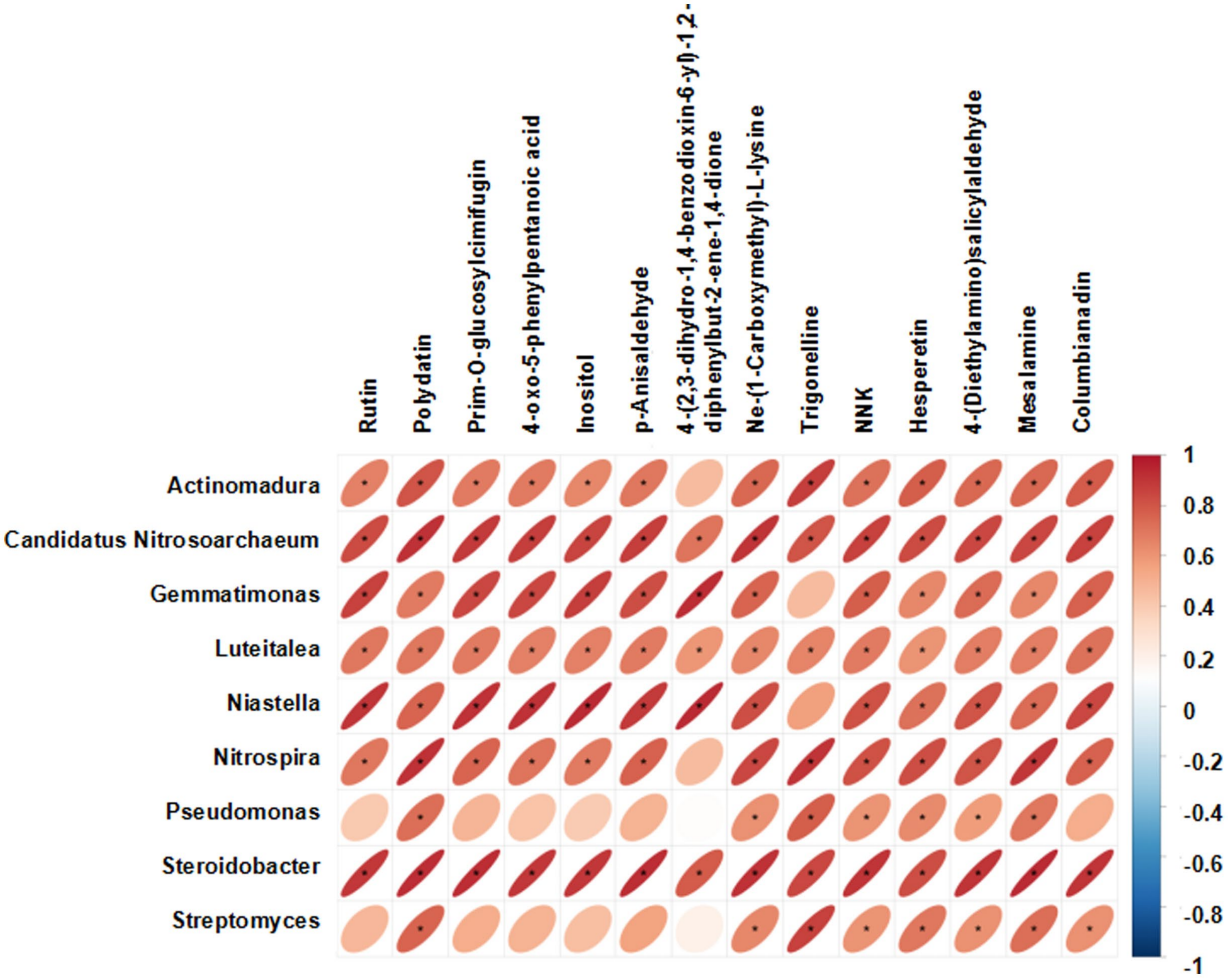
Pearson correlation test of the highly abundant communities and upregulated DAMs.

To gain a better understanding of the relationship of the bacterial communities and the metabolites in different cultivars, the Pearson correlation test of the highly abundant communities and upregulated DAMs was performed. There were 109 significant correlation combinations between the bacterial communities and metabolites that had a *p* value < 0.05 (Figure 9). The abundance of *Candidatus Nitrosoarchaeum, Luteitalea and Steroidobacter* were significantly positively correlated with all the metabolites.

## 4 Discussion

Phenols and flavonoids were found to be the main bioactive constituents in SYQ. There were significant differences among different cultivars in terms of bioactive constituents. As shown in Figure 1, the FC and TPC were higher in SYQ2 and SYQ4, compared to SYQ1 and SYQ3.

Before sampling, the four cultivars of SYQ were cultivated in the same soils for 3 years. It has been reported that the soil quality and plant health are strongly affected by the composition of bacterial community in soil (Berendsen et al., 2012). In this study, the rhizosphere community of different SYQ cultivars were investigated. The rhizospheres of grown cultivars showed different microbial composition and soil characteristics. Moreover, different accumulation patterns of metabolites were found in the roots of different cultivars. Cultivars with same bioactivities showed the similar metabolite accumulation trend. Previously, the microbial communities in the rhizosphere soils of different cultivars of *Brassica parachinensis* L. were significantly different (Huang et al., 2017). Similar findings were reported for cucumber cultivars (Zhou et al., 2015). These findings indicate that the plant cultivar is one of the main factors influencing the composition of soil bacteria.

SYQ cultivars exhibited differences in terms of dominant bacterial taxa in the rhizosphere bacterial communities. These dominant taxa in different SYQ cultivars may be involved in the growth of SYQs. Tuber size strongly correlated with bacterial community in SYQ (Hong et al., 2021). Significant differences in the composition of bacterial communities were observed among the four SYQ cultivars. In SYQ2 and SYQ4, *Actinomadura, Streptomyces, Pseudomonas, Nitrospira, Steroidobacter, Luteitalea, Candidatus Nitrosoarchaeum, Niastella* and *Gemmatirosa* were present in higher abundance, as compared to the other two cultivars.

*Streptomyces* and *Actinomadura* are Gram-positive bacteria belonging to Actinobacteria phylum. The addition of Actinobacteria decreased the pathogen incidence rate in plants (Contreras-Cornejo et al., 2016). *Streptomyces* is a rock-weathering bacteria, which is helpful in improving the nutrition conditions and plant growth (Zhang et al., 2017). There were some genes encoding polyketide and non-ribosomal peptide synthetases in the genome of Actinobacteria phyla. These bacteria have been reported to be involved in creating the suitable environment during plant growth (Calvaruso et al., 2006; Uroz et al., 2009). *Pseudomonas* and *Steroidobacter* belongs to phylum Proteobacteria. Proteobacteria are found abundantly in the rhizospheres of diverse plants and are involved in maintaining the soil ecological stability (Ren et al., 2020). The abundance of Gemmatimonadetes showed a positive correlation with soil moisture content. *Nitrospira* are nitrite-oxidizing bacteria, which play important roles in nitrogen cycle (Daebeler et al., 2020). *Candidatus nitrosoarchaeum* is an ammonia-oxidizing microorganism, the main ammonia oxidizers in many ecosystems (Mosier et al., 2012). Some nitrogen fixing bacteria were involved in the determination the number and yield of tuber of tuberous plants (Gururani et al., 2013).

The main metabolites in SYQ were found to be flavonoids and polyphenolic acids. Numerous studies have confirmed that some kaempferol and other flavonoids are associated with various biological activities. In this study, upregulation of rutin and hesperetin was observed in SYQs with higher bioactivities, indicating that they compose a large proportion of total bioactive compounds and can increase bioactivity of SYQ for various pharmaceutical purposes. In accordance with this assumption, we found they were the main bioactive compounds in both SYQ leaves and roots. Rutin is a flavonol glycoside which has potent antioxidant activity. Rutin has been reported to possess a range of pharmacological activities, including cytoprotective and neuroprotective activities (Scalbert et al., 2005) and antimicrobial and antiviral activity (Aditya and Ajay, 2017). Similarly, hesperetin is a flavanone glycoside and is commonly found in fruits, herbs and other plants (Garg et al., 2001). Hesperetin possesses antioxidant, anti-inflammatory, and cardioprotective properties (Parhiz et al., 2015). It was also demonstrated to augment the antioxidant cellular defenses via the ERK/Nrf2 signaling pathway and attenuate tissue damage induced by various compounds such as hydrogen peroxide (Parhiz et al., 2015). The differences observed in the bacterial community would influence the accumulation pattern of rutin and hespertin, thus affecting the FC, TPC and ESEC in SYQ roots.

## 5 Conclusion

In this study, rhizosphere soil bacterial community and the metabolites profiles of four SYQ cultivars grown in same soil were investigated. The rhizosphere bacterial community of different cultivars varied significantly. The findings suggest possible association between the dominance of some bacterial taxa and the metabolite profiles of SYQ roots. The upregulation of rutin and hesperetin may contribute to the high FC and TPC content in SYQ. The study indicates that certain bacterial species can be applied to increase the bioactivity of SYQ and special microbial fertilizer can be developed.

## Data availability statement

The datasets presented in this study can be found in online repositories. The names of the repository/repositories and accession number(s) can be found at: China National Microbiology Data Center (NMDC), NMDC40042549-NMDC40042560.

## Author contributions

YH: Conceptualization, Data curation, Writing – original draft. HH: Writing – review & editing. EY: Writing – review & editing. WY: Writing – review & editing. TN: Writing – review & editing. JY: Writing – review & editing. QL: Writing – review & editing. SR: Conceptualization, Writing – review & editing.

## Footnotes

^1^ http://topaz.gatech.edu/GeneMark/

^2^ http://www.bioinformatics.org/cd-hit/

^3^ https://github.com/bbuchfink/diamond/

^4^ http://www.hmdb.ca

^5^ http://metlin.scrippps.edu

^6^ http://kegg.jp
